# Effects of Carbidopa Premedication on ^18^F-FDOPA PET Imaging of Glioma: A Multiparametric Analysis

**DOI:** 10.3390/cancers13215340

**Published:** 2021-10-24

**Authors:** Marie Bros, Timothée Zaragori, Fabien Rech, Marie Blonski, Gabriela Hossu, Luc Taillandier, Pierre-Yves Marie, Antoine Verger

**Affiliations:** 1 Department of Nuclear Medicine and Nancyclotep Imaging Platform, CHRU-Nancy, Université de Lorraine, F-54000 Nancy, France; marie.bros12@hotmail.fr (M.B.); timothee.zaragori@univ-lorraine.fr (T.Z.); py.marie@chru-nancy.fr (P.-Y.M.); 2 IADI UMR 1254, INSERM, Université de Lorraine, F-54000 Nancy, France; g.hossu@chru-nancy.fr; 3 Department of Neurosurgery, CHRU-Nancy, Université de Lorraine, F-54000 Nancy, France; f.rech@chru-nancy.fr; 4 Centre de Recherche en Automatique de Nancy CRAN UMR 7039, CNRS, Université de Lorraine, F-54000 Nancy, France; m.blonski@chru-nancy.fr (M.B.); l.taillandier@chru-nancy.fr (L.T.); 5 Department of Neuro-Oncology, CHRU-Nancy, Université de Lorraine, F-54000 Nancy, France; 6 CIC 1433 Innovation Technologique, INSERM, Université de Lorraine, CHRU Nancy, F-54000 Nancy, France; 7 DCAC UMR 1116, INSERM, Université de Lorraine, F-54000 Nancy, France

**Keywords:** DOPA, PET, carbidopa, glioma, dynamic, radiomics

## Abstract

**Simple Summary:**

^18^F-FDOPA PET imaging is routinely used and recommended to assess gliomas. Carbidopa is a peripheral enzyme inhibitor. Carbidopa premedication increases the radiotracer uptake on static images. None of the evidence-based data available to date recommends carbidopa premedication. Our study therefore determined the impact of carbidopa premedication on static, radiomics and dynamic parameters for ^18^F-FDOPA PET brain tumor imaging. We show that carbidopa premedication leads to higher SUV and TTP dynamic parameters and impacts SUV-dependent radiomics by the same magnitude in healthy brains and tumors. The carbidopa effect is therefore compensated for by correcting for the tumor-to-healthy-brain ratio, a significant advantage for harmonizing data for multicentric studies. Results were obtained from simulations of time-activity curves using compartmental modeling.

**Abstract:**

Purpose: This study aimed to determine the impact of carbidopa premedication on static, dynamic and radiomics parameters of ^18^F-FDOPA PET in brain tumors. Methods: The study included 54 patients, 18 of whom received carbidopa, who underwent ^18^F-FDOPA PET for newly diagnosed gliomas. SUV-derived, 105 radiomics features and TTP dynamic parameters were extracted from volumes of interest in healthy brains and tumors. Simulation of the effects of carbidopa on time-activity curves were generated. Results: All static and TTP dynamic parameters were significantly higher in healthy brain regions of premedicated patients (ΔSUV_mean_ = +53%, ΔTTP = +48%, *p* < 0.001). Furthermore, carbidopa impacted 81% of radiomics features, of which 92% correlated with SUV_mean_ (absolute correlation coefficient ≥ 0.4). In tumors, premedication with carbidopa was an independent predictor of SUV_mean_ (ΔSUV_mean_ = +52%, *p* < 0.001) and TTP (ΔTTP = +24%, *p* = 0.025). All parameters were no longer significantly modified by carbidopa premedication when using ratios to healthy brain. Simulated data confirmed that carbidopa leads to higher tumor TTP values, corrected by the ratios. Conclusion: In ^18^F-FDOPA PET, carbidopa induces similarly higher SUV and TTP dynamic parameters and similarly impacts SUV-dependent radiomics in healthy brain and tumor regions, which is compensated for by correcting for the tumor-to-healthy-brain ratio. This is a significant advantage for multicentric study harmonization.

## 1. Introduction

L-3,4-dihydroxy-6-^18^F-fluoro-phenyl-alanine (^18^F-FDOPA) is a PET amino acid radiotracer that has been used to assess gliomas for over 20 years [1]. The PET-RANO group (Response Assessment in Neuro-Oncology) recommends its use at the primary diagnosis, for monitoring disease and therapy, and for diagnosing tumor recurrence [2,3,4]. 

In addition to its physiological uptake in the striatum,^18^F-FDOPA has a relatively high specificity for gliomas, conferred by its ability to cross an intact blood-brain barrier and the overexpression of Large Amino acid Transporters (LATs) in tumors [5,6], making it a useful adjunct to contrast-enhanced brain MRI, which remains the gold standard for the diagnostic assessment of gliomas.

Carbidopa (L-α-hydrazino-α-methyl-β-(3,4-dihydroxyphenyl)propionic acid) is a peripheral inhibitor of aromatic amino acid decarboxylase. Its use as a premedication therefore results in higher plasma concentrations of ^18^F-FDOPA and of its metabolite ^18^F-OMFD (3-O-methyl-6-[^18^F]fluoro-L-DOPA) [7]. As the ^18^F-FDOPA transport rate constant from plasma to tumor cells via LAT transporters, K1 [8], and the net influx rate constant, Ki [7], are not affected by this premedication, carbidopa pretreatment leads to higher radiotracer uptake in both healthy brains and glioma [9]. The proportion of uptake increases in these two structures, and the effects of carbidopa premedication on the radiomics parameters as well as the dynamic analysis nevertheless remain to be determined. To date, only one study consisting of two patients premedicated with 200 mg of carbidopa showed an average 50% higher uptake in the cerebellum, striatum and the tumor, based on acquisitions obtained 15 to 25 min post-injection [9].

In contrast to movement disorder PET imaging [10], international guidelines do not recommend administering carbidopa before ^18^F-FDOPA PET for brain tumor imaging, solely based on the fact that most of the published studies do not use it. There is currently no evidence-based data to determine whether carbidopa should be used in the clinical setting of brain tumor imaging, particularly in the current era of routine semi-quantitative analyses [3].

The objective of this study is therefore to determine the impact of carbidopa premedication on static, radiomics and dynamic parameters in brain tumor ^18^F-FDOPA PET imaging.

## 2. Materials and Methods

### 2.1. Population and ^18^F-FDOPA PET Imaging

We retrospectively selected newly diagnosed gliomas, which were all classified or re-classified according to the WHO 2016 classification [11]. All newly diagnosed glioma patients had undergone an ^18^F-FDOPA PET at the CHRU of Nancy between January 2013 and October 2017. Carbidopa premedication data were available for all patients included in the study and depended on the routine examination protocol performed; patients analyzed from March 2016 to October 2017 were premedicated with carbidopa, and patients analyzed from January 2013 to February 2016 were not premedicated prior to PET imaging. The data evaluation process was approved by the local ethics committee (Comité d’Éthique du CHRU de Nancy) on 26 August 2020. The trial was registered at ClinicalTrials.gov (NCT04469244). This research complied with the principles of the Declaration of Helsinki. Informed consent was obtained from all individuals included in the study. 

^18^F-FDOPA PET-computed tomography (CT) scans were performed on a Biograph hybrid system involving a six-detector CT for attenuation correction (Biograph 6 True Point, SIEMENS, Erlangen, Germany). All patients were instructed to fast for at least 4 h, and patients analyzed from March 2016 to October 2017 received 100 mg of carbidopa 1 h prior to their examination. A CT scan was first recorded for each patient, immediately followed by a 30 min 3D list mode PET recording, initiated during the bolus injection of 3 MBq of ^18^F-FDOPA per kilogram of body weight. Static PET images were reconstructed from the list mode data acquired 10 to 30 min post-injection, while dynamic PET images consisted of 30 frames of one minute each [12]. Static and dynamic images were reconstructed using the OSEM 2D algorithm (2 iterations, 21 subsets, 4 mm Gaussian post-reconstruction filter, 256 × 256 × 148 voxels of 2.7 × 2.7 × 3.0 mm^3^). All images were corrected for attenuation using CT, dead time, random and scattered coincidences during the reconstruction process.

### 2.2. Image Analyses

#### 2.2.1. Segmentation

The LIFEx software (lifexsoft.org) was used to define volumes of interest (VOIs) for tumors and contralateral healthy brains [13]. 

To measure healthy brain uptake, patient-specific crescent-shaped contours for the healthy brain VOI were drawn on three consecutive image slices and encompassed both white and grey matter on the semi-oval center of the unaffected hemisphere, as previously recommended [14].

In tumors, VOIs were segmented semi-automatically using a threshold of 1.6 healthy brain SUV_mean_ [15]. For tumors with multiple loci, we only considered the site on which the neuropathological diagnosis was performed. All final VOIs were visually inspected by an experienced physician (A.V.) to ensure that the quality of the methods applied was consistent throughout and to exclude any potentially pathological areas detected on the MRI (oedema or any other intercurrent pathology) from the VOIs defined as healthy brains. 

#### 2.2.2. Extraction of Parameters

For static images, the SUV_mean_, SUV_max_ and SUV_peak_ parameters were extracted from the previously described VOIs for healthy brains and tumors. SUV_peak_ was defined as the highest mean uptake in a 1 cm^3^ sphere, centered on each voxel of the VOI.

For the radiomics analysis, 105 features were extracted from the same brain and tumor VOIs. These included morphological, local intensity, intensity-based statistical, intensity histogram and textural parameters. In accordance with the guidelines and benchmark values of the image standard biomarker initiative [16], 103 parameters were extracted using PyRadiomics, and 2 local-intensity parameters, which were not available on PyRadiomics, were extracted with in-house software [12]. These radiomics parameters were extracted as detailed elsewhere [12]. Briefly, isotropic voxel resampling was performed using tricubic spline interpolations, before carrying out an absolute discretization of PET intensities with a fixed bin size of 0.1. Parameters were computed from a single matrix after merging all 3D directional matrices.

To potentially correct for any carbidopa premedication effects in our population, all static and radiomics parameters, except morphological features, in tumors were re-extracted after normalizing each static image for the SUV_mean_ of healthy brain VOI, to compute the Tumor-to-normal-Brain Ratio (TBR) parameters. 

To take into account any potential patient movement during the dynamic acquisition, each dynamic frame was first registered to the associated CT image [17]. The SUV_mean_ values for each frame were respectively computed in the brain VOI and in the VOI corresponding to the tumor SUV_peak_ on the static image to extract the brain and tumor time activity curves (TACs). TACs were fitted to overcome noise effects [12]. As previously defined, two dynamic parameters were extracted: time-to-peak (TTP) and slope [18]. 

As for parameters extracted from static images, a normalized version of the parameters was extracted from a TAC, representing the evolution of the ratio between tumor and brain fitted TACs to potentially correct for any carbidopa premedication effect [12].

### 2.3. Simulation

To indirectly confirm our hypotheses and compare the effects of carbidopa premedication on dynamic analysis in the same brain tumor of patients who underwent ^18^F-FDOPA PET, both without and with premedication, simulations were performed. Simulated TACs of the carbidopa premedication status were computed based on the TACs of 15 patients without premedication, for which raw data were available (median age 58.5 (45.2; 64.1) years, 5 women). Five patients were classified as IDH mutant astrocytomas (3 anaplastic), 4 as IDH wildtype astrocytomas (2 anaplastic), 2 as IDH mutant and 1p/19q co-deleted oligodendrogliomas (1 anaplastic) and 4 as IDH wildtype glioblastomas.

**Figure 1:**
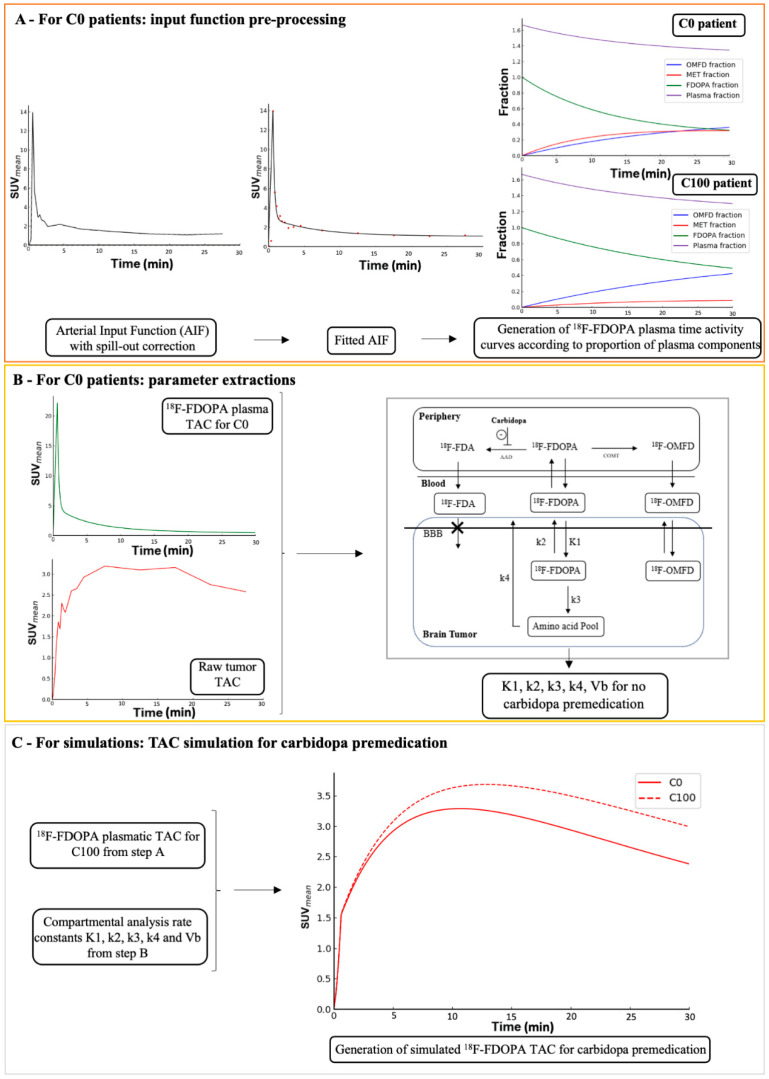
Generation of simulated time-activity curves simulating carbidopa premedication in the tumor volumes of interest (VOIs). The same processes were applied to healthy brain VOIs. (**A**) For C0 patients: input function pre-processing; (**B**) For C0 patients: parameter extractions; (**C**) For simulations: TAC simulation for carbidopa premedication.

This was performed using a compartmental model analysis. Dynamic PET images were reconstructed using 26 frames of 8 × 15 s, 2 × 30 s, 2 × 60 s, and 5 × 300 s [9]. Total blood TACs of patients having undergone ^18^F-FDOPA PET imaging without any premedication were extracted from VOIs placed into the internal carotid by an experienced physician (A.V.), using initial dynamic frames to identify early vascular phases [19]. The spill-out coefficient was estimated at 0.35 by simulation and used to correct from partial volume effect on blood TACs [20]. These blood TACs were then fitted to the peak, using linear interpolation followed by a tri-exponential function after the peak [21]. Plasma ^18^F-FDOPA input functions were computed after metabolite correction, according to the methodology described by Wardak et al. [22] and by using previously published values of metabolite proportions of hematocrit, as follows: (i) proportions of each of the ^18^F-labeled metabolites determined by Melega et al. [23] were used as they included the proportions for no premedication as well as for premedication with 100 mg of carbidopa (similarly to ours) (respectively C0 and C100 in Figure 1A) and (ii) a mean 40% hematocrit value was selected for each patient, as previously reported [8]. The compartmental model used for tumors was a two-tissue compartmental model validated for compartmental analysis of ^18^F-FDOPA PET imaging in gliomas [22], as it satisfied the minimum Akaike information criterion when compared to a simpler one-tissue compartmental model [24] or to a two-tissue compartmental model with fewer parameters (V_b_ fixed at 5% [8] or k_4_ set to 0).

Raw TACs for healthy brains and tumors were extracted from the dynamic analysis of the 15 patients that had undergone ^18^F-FDOPA PET imaging without any premedication. Compartmental models were fitted to these TACs using the previously described input function to obtain the four rate constants (K_1_, k_2_, k_3_, k_4_) as well as the blood volume fraction (V_b_) (Figure 1B). For the simulation of the carbidopa premedication status of brain and tumor TACs, the four rate constants (K_1_, k_2_, k_3_, k_4_) were assumed to be unchanged compared to those calculated for the patients without any premedication, given that the ^18^F-FDOPA K1 [8] and the net influx rate constant Ki [7] are not affected by the premedication. The carbidopa premedication status of healthy brain and tumor TACs were then simulated using the plasma ^18^F-FDOPA input function corresponding to the carbidopa premedication (C100, Figure 1C). 

TTP and slope were extracted from healthy brain and tumor TACs for the patients as well as for the simulated TACs. A normalized version of these parameters was also extracted.

### 2.4. Statistical Analysis

Categorical variables are expressed as percentages and continuous variables as medians and interquartile ranges. Intergroup comparisons were performed with the Chi-squared test for categorical variables and the Mann-Whitney test for continuous variables. Mann-Whitney tests were performed to compare carbidopa-naïve and premedicated patients in healthy brain VOIs. Correlations between radiomics features and SUV_mean_ were assessed using the Spearman correlation coefficient. Correction of multiples tests was performed with the Benjamini-Hochberg correction, and *p* < 0.05 was considered significant. For tumor VOIs, linear regression analyses were performed to predict the parameters using carbidopa status and histo-molecular diagnosis as covariates, as histo-molecular diagnosis is known to influence static and dynamic parameters (gliomas were classified as IDH-wildtype and IDH-mutant astrocytomas, IDH-mutant and 1 p/19 q co-deleted oligodendrogliomas, and IDH-wildtype and IDH mutant glioblastomas) [18]. The significance of each covariate was tested using a type III analysis of variance. Analyses were performed with the R software version 3.6.2 (R Foundation for Statistical Computing, Vienna, Austria) and Python (Python Software Foundation). 

## 3. Results 

### 3.1. Patient Characteristics 

Fifty-four patients with a median age of 44 (19.8; 82.6) years, comprised of 19 (35%) women and 18 (33%) patients who were premedicated with 100 mg of carbidopa, were included in the study. Dynamic acquisitions were available for 41 of these patients (median age of 45.2 (19.8; 73.7) years, 14 (34%) women, 11 (27%) patients premedicated with carbidopa). Detailed patient characteristics are provided in Table 1. 

### 3.2. Carbidopa Effects

In this study, carbidopa induced higher values for all static and dynamic parameters in the brain compared to patients that were not premedicated with carbidopa, with ΔSUV_mean_ = +53%, ΔTTP = +48% and Δslope = +88% (all with *p* < 0.001) (Table 2). Furthermore, our results showed that in healthy brains, 81% of radiomics features were impacted by carbidopa, and 92% of these correlated with SUV_mean_ (absolute correlation coefficient ≥ 0.4) (Supplementary Figure S1).

In tumors, carbidopa premedication was an independent predictor of SUV_mean_ (ΔSUV_mean_ = +52%, *p* < 0.001) and TTP (ΔTTP = +24%, *p* = 0.025). Histo-molecular diagnosis was predictive of TTP (*p* = 0.010) and slope (*p* < 0.001), and a trend was observed for the SUV_mean_ (*p* = 0.07). Interestingly, all static, dynamic and radiomics parameters were no longer significantly modified by carbidopa premedication when using tumor-to-healthy-brain image ratios or time-activity curve ratios (Table 3 and Supplementary Tables S1–S4).

**Figure 2:**
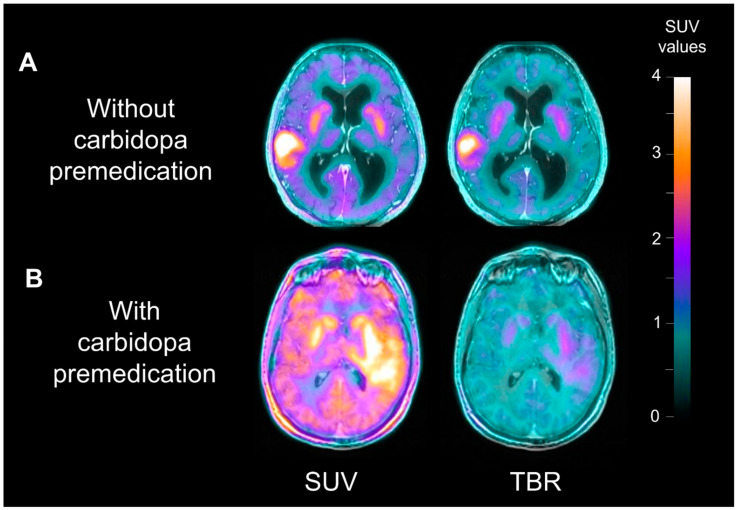
Representative examples of two patients with or without carbidopa premedication, before and after using tumor-to-healthy-brain ratio parametric images. Patient (**A**) is a 66-year-old woman with a newly diagnosed IDH wildtype glioblastoma (SUV_mean_ 3.2). Patient (**B**) is a 32-year-old man with a newly diagnosed IDH wildtype glioblastoma (SUV_mean_ 4.1). The two tumors are clearly different on TBR-parametric images, even though they have essentially the same SUV values on SUV-parametric images. Note the higher SUV uptake of striatal and healthy brain areas on B due to the carbidopa premedication.

Representative examples of glioblastoma IDH wildtype patients with or without carbidopa premedication before and after using tumor-to-healthy-brain ratio (TBR) parametric images are shown in Figure 2.

To confirm our hypotheses about the impact of carbidopa premedication on TTP and to better understand its effect on slope, simulated TACs were performed with carbidopa premedication, on the assumption that carbidopa premedication induces an increase of radiotracer availability through the input function, i.e., the plasma concentration of ^18^F-FDOPA, without modifying the rate constants. Results of these simulations are provided in Table 4.

**Figure 3:**
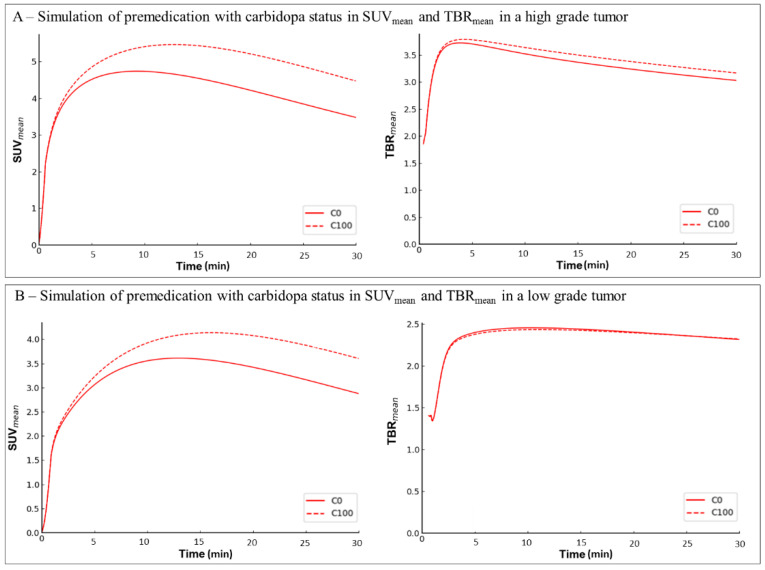
Typical examples of patients without carbidopa premedication (C0) simulated with the effects of 100 mg carbidopa premedication (C100). (**A**) ^18^F-FDOPA PET time-activity curves expressed as SUV_mean_ (left panel) of a 59-year-old woman with IDH wildtype glioblastoma. Carbidopa induces a longer TTP (TTP C0 = 9 mn; TTP C100 = 12.8 mn) and a higher slope (slope C0 = −4 SUV/h; SUV C100 = −3.1 SUV/h). ^18^F-FDOPA PET time-activity curves expressed in TBR_mean_ (right panel). The differences of TTP and slope values between carbidopa statuses are reduced when using Tumor-to-normal-Brain ratio (TBR) images (TTP C0 = 3.8 mn vs. TTP C100 = 4.2 mn; slope C0 = −1.45 SUV/h vs. slope C100 = −1.4 SUV/h); (**B**) ^18^F-FDOPA PET time-activity curves expressed as SUV_mean_ (left panel) of a 65-year-old man with oligodendroglioma. Carbidopa induces a longer TTP (TTP C0 = 13.6 mn; TTP C100 = 17.2 mn) and a higher slope (slope C0 = −0.7 SUV/h; SUV C100 = −0.3 SUV/h). ^18^F-FDOPA PET time-activity curves expressed as TBR_mean_ (right panel). The differences of TTP and slope values between carbidopa statuses are reduced when using Tumor-to-normal-Brain ratio (TBR) images (TTP C0 = 10.4 mn vs. TTP C100 = 11 mn; slope C0 = −0.45 SUV/h vs. slope C100 = −0.4 SUV/h).

Examples of simulated carbidopa premedication leading to higher TTP are displayed in Figure 3.

## 4. Discussion

This study shows that carbidopa premedication before ^18^F-FDOPA PET imaging of brain tumors is associated with higher SUV, SUV-related radiomics and TTP dynamic parameters, of the same order of magnitude as in healthy brains. For neuro-oncological PET indications, the effect of the costly and time-consuming carbidopa premedication is thus limited when using TBR images and TAC ratios, which are efficient tools for multicentric study harmonization. 

Carbidopa premedication in the present study leads to an approximately 50% higher SUV_mean_ in healthy brain tissue as well as and in brain tumors (Table 2 and Table 3). To the best of our knowledge, to date, only one previously published study evaluated the effects of carbidopa in brain tumors, by comparing two patients with stable brain tumors, premedicated with 200 mg of carbidopa at baseline, and reevaluating these same patients without premedication one year later. Consistent with our study, this previous study also reported a 50% higher uptake in all brain structures, including healthy brain and tumors from acquisitions performed 15 to 25 min post-injection [9]. 

Our current study found that 81% of all radiomics parameters in healthy brains were significantly modified by carbidopa premedication, among which 92% correlated with SUV_mean_ (85 parameters, absolute correlation coefficient ≥ 0.4 in 78 parameters) (Supplementary Figure S1). This underlines the fact that the effect of carbidopa premedication is related to a relatively homogeneous higher uptake of the ^18^F-FDOPA radiotracer within the VOIs. The impact of SUV values on textural features has already been highlighted by Orlhac et al. [25]. As we are using absolute discretization here, the higher SUV related to carbidopa premedication induced two concomitant observations: (i) a shift of textural matrices to higher bin values (bin shift), mainly responsible for the modification of the parameters correlated with the SUV values and (ii) a relative spread of the distribution of SUV values over a larger number of bins (bin spread). Eight percent of radiomics features (seven parameters, Supplementary Figure S1) were significantly modified by carbidopa premedication but poorly correlated with SUV_mean_ values (absolute correlation coefficients ranging from 0.27 to 0.34). Beyond the impact of a threshold effect, we presume that these parameters are less correlated with SUV_mean_ because they mainly depend on the bin spread effect, for which the impact on radiomics parameters is less directly correlated with SUV_mean_ than the bin shift effect. The bin spread effect on the involved matrices is illustrated in Supplementary Figure S2. Among the 20 remaining parameters, most are not significantly modified by carbidopa premedication, since they are not correlated with SUV_mean_ values (Supplementary Figure S1). 

In our study, carbidopa also induced changes in the dynamic analysis parameters, with higher TTP in healthy brains and in tumors. In tumors, this effect was independent of the histo-molecular diagnosis, which is known to affect the TTP of the dynamic analysis [18,26,27,28]. Even if the slope parameter was influenced by the histo-molecular diagnosis, no significant predictive value of carbidopa premedication was observed in tumors, while carbidopa induced significantly higher slope in healthy brains. To confirm our findings in patients, we performed simulations on the assumption that carbidopa premedication leads to an increase in the plasma availability of ^18^F-FDOPA without modifying the rate constants (Figure 1). These simulations confirm that the higher TTP was related to carbidopa premedication, similar to what was observed in patients. Moreover, a higher slope was also observed in the simulated data. It is of concern that the higher TTP due to carbidopa premedication could potentially lead to an underestimation of tumor aggressiveness, which stresses the importance of harmonizing heterogenous premedication data. In fact, this probability is moderate with regard to the small degree of variation observed in the absolute values of TTP and slope in the tumors after simulation of carbidopa premedication (around +4 min and +0.5 SUV/h, respectively). 

Interestingly, when extracted from TBR images and TAC ratios, all static, dynamic and radiomics parameters were no longer significantly modified by the carbidopa premedication (Table 3 and Table 4 and Supplementary Tables S1–S4). This is an important observation, as TBRs are typically implemented in the routine analysis of neuro-oncology scans. These ratios were used in most articles in the literature for static [29,30], dynamic [18] and radiomics [12] parameters, but without having been yet validated as to the effect of the carbidopa premedication. 

Glioma is a rare pathology, which relies on multicentric studies to gather high enough patient numbers. Such multicentric studies can only be conducted after harmonizing data obtained from the participating centers. Although the use of carbidopa premedication is very heterogenous between individual centers, the harmonization process may only entail using TBR images and TAC ratios, which have been shown to be insensitive to carbidopa premedication in this study, without modifying the protocols used by participating centers. While the ^18^F-FDOPA PET guidelines for imaging of Parkinsonian syndromes [10] recommend carbidopa premedication to increase the systemic and thus central nervous system availability of ^18^F-FDOPA, this recommendation is completely different in brain tumors, given that, unlike striatum, brain tumor cells do not metabolize ^18^F-FDOPA [9]. This is presumably why recommendations for ^18^F-FDOPA PET imaging do not include mandatory carbidopa premedication prior to brain tumor scans. This recommendation is however not founded on any evidence-based data from the literature, and our study therefore provides additional information that supports the current recommendation. It should be noted that premedication with Carbidopa before PET imaging increases ^18^F-FDOPA bioavailability, leading to higher uptake and thus improved image quality. This was a particularly significant issue in older low-sensitivity PET scanners and is therefore a problem encountered in studies that use retrospective data. Today’s state-of-the-art high-resolution scanners have eliminated this issue. This means that the only remaining rationale for using carbidopa premedication would be to reduce patients’ radiation exposure to an absolute minimum, which is not a key concern in glioma patients, for whom radiotherapy is one of the treatments of choice.

Our retrospective study was performed on a heterogeneous population of patients, therefore limiting the ability to directly compare the effects of carbidopa premedication in tumors. Our hypotheses on the effects of carbidopa premedication were nevertheless confirmed by using simulated data. In our department, the patient carbidopa status was predicated by the recruitment period; this may have introduced an inclusion bias. However, no significant differences were observed between the characteristics of the two groups of patients (Table 1). Finally, ^18^F-FDOPA and premedication with carbidopa are not ubiquitously available in all parts of the world, with other PET tracers also yielding excellent performances in brain tumor detection [31].

## 5. Conclusions

Our current study documents the effects of carbidopa premedication on the ^18^F-FDOPA PET imaging of brain tumors. As carbidopa premedication leads to higher SUV, SUV-dependent radiomics and TTP dynamic parameters in the same order of magnitude in healthy brains as in tumor, these effects are compensated for after taking into account the tumor-to-healthy-brain ratios in static images or in time-activity curves, which is an important point for multicentric study harmonization.

## Figures and Tables

**Table 1:** Patient characteristics.

	Without Carbidopa	With Carbidopa	All Patients	*p* Value
	Premedication	Premedication		
*n*				
Static	36	18	54	
Dynamic	30	11	41	
Age (years) median (range)				
Static	45.2 (24.6; 82.6)	37.4 (19.8; 72.7)	44.0 (19.8; 82.6)	0.21
Dynamic	46.7 (24.6; 73.7)	33.3 (19.8; 72.8)	45.2 (19.8; 73.7)	0.13
Female gender *n* (%)				
Static	11 (31%)	8 (44%)	19 (35%)	0.48
Dynamic	10 (33%)	4 (36%)	14 (34%)	1
Primary histopathological type *n* (%)				
Static				
IDH-mutant astrocytoma				
Anaplastic	13 (36%)	3 (17%)	16 (30%)	
Non-anaplastic	4	1	5	
IDH-wildtype astrocytoma	9	2	11	
Anaplastic	6 (17%)	1 (6%)	7 (13%)	
Non-anaplastic	3	1	4	0.21
IDH-mutant and 1p/19q co-deleted	3	0	3	
oligodendroglioma	9 (25%)	5 (28%)	14 (26%)	
Anaplastic				
Non-anaplastic	6	2	8	
IDH-wildtype glioblastoma	3	3	6	
IDH-mutant glioblastoma	6 (17%)	6 (33%)	12 (22%)	
	2 (6%)	3 (17%)	5 (9%)	
Dynamic				
IDH-mutant astrocytoma				
Anaplastic	13 (43%)	2 (18%)	15 (37%)	
Non-anaplastic	4	1	5	
IDH-wildtype astrocytoma	9	1	10	
Anaplastic	4 (13%)	0 (0%)	4 (10%)	0.17
Non-anaplastic	2	0	2	
IDH-mutant and 1p/19q co-deleted	2	0	2	
oligodendroglioma	7 (23%)	4 (36%)	11 (27%)	
Anaplastic				
Non-anaplastic	5	2	7	
IDH-wildtype glioblastoma	2	2	4	
IDH-mutant glioblastoma	5 (17%)	5 (45%)	10 (24%)	
	1 (3%)	0 (0%)	1 (2%)	

Note: *p*-value for comparing patients with and without carbidopa premedication.

**Table 2:** Healthy brain extracted parameters in patients without and with carbidopa premedication.

Parameter	Without Carbidopa Premedication	With Carbidopa Premedication	Correlation Coeffi-cient with SUV_mean_	*p* Value	Mean Relative Difference in % (Mean Absolute Difference) between Patients without and with Carbidopa Premedication	Median Relative Difference in % (Median Absolute Difference) between Patients without and with Carbidopa Premedication
**Static features (in-house software)**						
SUVmean	1.2 (1.02; 1.32)	1.8 (1.53; 2.08)	**1**	**<0.001**	53.1% (+0.6)	46.4% (+0.5)
SUVmax	1.6 (1.45; 1.75)	2.4 (2.03; 2.68)	**0.99**	**<0.001**	48% (+0.8)	41.3% (+0.7)
SUVpeak	1.4 (1.27; 1.57)	2.1 (1.8; 2.36)	**0.99**	**<0.001**	50.2% (+0.7)	44.2% (+0.6)
**Dynamic features (in-house software)**						
TTP (min)	12.5 (10.74; 13.64)	18.5 (17.03; 18.96)	**0.61**	**<0.001**	48.5% (+6.1)	46.2% (+5.6)
Slope (SUV/h)	−0.52 (−0.69; −0.39)	−0.06 (−0.18; 0.01)	0.35	**<0.001**	87.8% (+0.5)	70% (+0.4)

Note: *p*-value for comparing patients without and with carbidopa premedication (in bold, significant *p*-values); absolute correlation coefficients ≥ 0.4 are in bold; TTP: time-to-peak; SUV: Standard Uptake Value.

**Table 3:** Brain tumor extracted parameters in patients without and with carbidopa premedication and after normalization for healthy brain parameters with Tumor-to-normal-Brain Ratios (TBRs).

Parameters	Without Carbidopa Premedication	With Carbidopa Premedication	Correlation Coefficient with SUV_mean_	*p* Value	Mean Relative Difference in % (Mean Absolute Difference) between Patients without and with Carbidopa Premedication	Median Relative Difference in % (Median Absolute Difference) between Patients without and with Carbidopa Premedication	TBR without Carbidopa Premedication	TBR with Carbidopa Premedication	TBR *p* Value
**Static features (in-house software)**									
SUV_mean_	2.4 (1.94; 2.8)	3.6 (3.14; 4.06)	**1**	**<0.001**	51.9% (+1.2)	48% (+1.1)	2.1 (1.78; 2.19)	2.1 (1.87; 2.15)	0.458
SUV_max_	4.1 (2.6; 5.15)	5.8 (4.08; 7.18)	**0.84**	**0.034**	41.4% (+1.7)	41.9% (+1.5)	3.5 (2.18; 4.26)	3.3 (2.44; 4.09)	0.291
SUV_peak_	3.4 (2.02; 4.21)	4.9 (3.62; 6.16)	**0.86**	**0.034**	44.1% (+1.5)	51.2% (+1.5)	2.9 (1.84; 3.49)	2.8 (1.98; 3.31)	0.335
MTV (mL)	20 (4.02; 29.07)	20.1 (5.79; 24.77)	0.38	0.608	0.9% (+0.2)	31.2% (+3.7)	20 (4.02; 29.07)	20.1 (5.79; 24.77)	0.608
**Dynamic features (in-house software)**							12.2 (2.83; 23.15)	10.3 (2.48; 18)	0.696
TTP (mn)	12.2 (7.37; 15.53)	15.1 (9.43; 19.31)	0.02	**0.025**	24.1% (+2.9)	30.3% (+3.1)	−1.1 (−1.59; 0.41)	−1.7 (−1.9; −0.02)	0.505
Slope (SUV/h)	−2.71 (−3.14; −0.62)	−3.18 (−3.67; 0.14)	-0.27	0.962	−17.6% (−0.5)	−93.8% (−1.1)	2.1 (1.78; 2.19)	2.1 (1.87; 2.15)	0.458

Note: *p*-value for the predictive value of the carbidopa premedication in a linear regression model, including histo-molecular diagnosis without and with Tumor-to-normal-Brain ratios (TBR) (in bold, significant *p*-values). Absolute correlation coefficients in bold are ≥ 0.4. MTV: Metabolic Tumor Volume; TTP: Time to Peak; SUV: Standardized Uptake Value.

**Table 4:** Brain tumor extracted dynamic parameters in simulations and after normalization for healthy brain parameters with Tumor-to-normal-Brain Ratios (TBRs).

Parameters	Without Carbidopa Premedication	With Carbidopa Premedication	Mean Relative Difference in % (Mean Absolute Difference) between Patients without and with Carbidopa Premedication	Median Relative Difference in % (Median Absolute Difference) between Patients without and with Carbidopa Premedication	TBR without Carbidopa Premedication	TBR with Carbidopa Premedication	Mean Relative Difference in % (Mean Absolute Difference) between Patients without and with Carbidopa Premedication	Median Relative Difference in % (Median Absolute Difference) between Patients without and with Carbidopa Premedication
**Dynamic features**								
TTP (mn)	10.7 (7.36; 12.75)	13.0 (11.13; 15.68)	25.8% (+2.9)	21.4% (+2.3)	4.6 (3.78; 9.27)	4.7 (3.92; 9.70)	2.9% (+0.3)	2.2% (+0.1)
Slope (SUV/h)	−2.98 (−3.93; −1.31)	−2.21 (−3.32; −0.85)	23.5% (+0.7)	25.9% (+0.8)	−1.3 (−1.81; −0.07)	−1.3 (−1.67; −0.11)	−0.7% (−0.1)	0% (0)

TBR: Tumor-to-normal-Brain ratio; TTP: Time to Peak.

## Data Availability

All data generated or analyzed during this study are included in this published article and its supplementary information files.

## Supplementary Materials

The following are available online at https://www.mdpi.com/article/10.3390/cancers13215340/s1, Figure S1: Effect of carbidopa premedication on healthy brain radiomics parameters, Figure S2: Illustration of bin spread effect on matrices in which some textural features were significantly modified with carbidopa, but poorly correlated to SUVmean, Table S1−S4: Brain tumor radiomic parameters extracted from patients without and with carbidopa premedication and after normalizing for healthy brain parameters with Tumor to normal Brain Ratios (TBR).
