# Supplementary material for: Effects of Carbidopa Premedication on 18F-FDOPA PET Imaging of Glioma: A Multiparametric Analysis
Source: Cancers (Basel). 2021 Oct 24;13(21):5340. doi: 10.3390/cancers13215340 (PMC8582429; doi:10.3390/cancers13215340)
Supplement: Supplementary file 1 [file cancers-13-05340-s001.zip › Supplementary_file_Cancers.pdf]

Supplemental Table S1. Brain tumor radiomic parameters extracted from patients without and with carbidopa premedication and after normalizing for healthy brain parameters with Tumor to normal Brain Ratios (TBR).

Parameters	Without carbidopa premedication	With carbidopa premedication	Correlation coefficient with SUV_{mean}	p value	Delta mean value in % (absolute value) between patients without and with carbidopa premedication	Delta median value in % (absolute value) between patients without and with carbidopa premedication	TBR Without carbidopa premedication	TBR With carbidopa premedication	TBR p value
**Morphological features (pyradiomics)**									
Volume (mesh)	19555.728 [3815.0;28696.7]	19904.328 [6750.8;24252.3]	0.38	0.622	+1.8% (348.6)	+26.0% (3964.4)	8876.463 [3815.0;28696.7]	10713.917 [6750.8;24252.3]	0.622
Volume (counting)	19883.864 [4047.9;28696.7]	20247.182 [7064.8;24688.7]	0.38	0.622	+1.8% (363.3)	+25.6% (3962.0)	9140.775 [4047.9;29061.2]	10991.843 [7064.8;24688.7]	0.622
Surface area (mesh)	4617.381 [1672.3;6606.9]	4817.326 [2465.1;5691.9]	0.35	0.634	+4.2% (199.9)	+10.5% (414.9)	2878.096 [1672.3;6606.9]	3266.035 [2465.1;5691.9]	0.634
Surface to volume ratio	0.399 [0.23;0.49]	0.341 [0.22;0.44]	**-0.42**	0.969	-17.1% (-0.05)	-2.4% (-0.007)	0.413 [0.23;0.49]	0.354 [0.22;0.44]	0.969
Sphericity	0.706 [0.62;0.79]	0.682 [0.58;0.78]	0.05	0.746	-3.5% (-0.02)	+3.5% (0.03)	0.71 [0.62;0.79]	0.715 [0.58;0.78]	0.746
Maximum 3D diameter	46.141 [29.7;59.3]	49.391 [41.86;55.21]	0.29	0.855	+6.6% (3.2)	+2.4% (1.2)	37.966 [29.73;59.35]	41.451 [41.86;55.21]	0.855
Major axis length	38.592 [24.4;50.4]	43.344 [33.65;50.28]	0.31	0.893	+11.0% (4.8)	+11.9% (5.4)	32.922 [24.43;50.38]	35.919 [33.65;50.28]	0.893
Minor axis length	27.998 [17.01;39.14]	31.613 [23.14;38.2]	0.38	0.900	+11.4% (3.6)	+15.5% (4.9)	24.136 [17.01;39.14]	27.193 [23.14;38.21]	0.900
Least axis length	21.013 [12.5;28.25]	21.936 [15.94;27.38]	0.37	0.587	+4.2% (0.9)	+10.6% (2.3)	17.363 [12.4;28.25]	18.494 [15.93;27.38]	0.587

Elongation	0.729 [0.63;0.86]	0.738 [0.66;0.80]	0.20	0.789	1.3% (0.01)	-6.1% (-0.04)	0.739 [0.63;0.86]	0.77 [0.66;0.80]	0.789
Flatness	0.55 [0.478;0.67]	0.509 [0.46;0.60]	0.08	0.267	-8.0% (-0.04)	-7.6% (-0.04)	0.548 [0.48;0.67]	0.533 [0.46;0.60]	0.267
**Local intensity features (in-house software)**									
Local intensity peak	3.321 [1.97;4.14]	4.732 [3.56;5.93]	**0.86**	**0.040**	+29.8% (1.4)	+32.8% (1.4)	2.872 [1.78;3.48]	2.723 [1.95;3.31]	0.314
Global intensity peak	3.406 [2.03;4.20]	4.9 [3.61;6.13]	**0.85**	**0.035**	+30,5% (1.5)	+33.6% (1.5)	2.942 [1.84;3.49]	2.811 [2.0;3.34]	0.339
**Intensity-based statistical features (pyradiomics)**									
Mean intensity	2.394 [1.93;2.80]	3.629 [3.14;4.05]	**0.99**	**< 0.001**	+34.0% (1.2)	+34.9% (1.3)	2.418 [1.78;2.19]	2.327 [1.85;2.15]	0.450
Intensity variance	0.365 [0.02;0.41]	0.573 [0.10;0.86]	**0.65**	0.719	+36.4% (0.2)	+68.9% (0.2)	0.215 [0.02;0.31]	0.148 [0.04;0.28]	0.304
Intensity skewness	0.995 [0.65;1.33]	0.953 [0.79;1.10]	0.02	0.616	-4.3% (-0.04)	-9.2% (-0.08)	0.843 [0.65;1.33]	0.799 [0.79;1.10]	0.616
(Excess) intensity kurtosis	0.727 [-0.19;1.39]	0.336 [-0.13;0.51]	-0.03	0.311	-116.6% (-0.4)	-101.9% (-0.3)	0.182 [-0.19;1.39]	-0.057 [-0.13;0.51]	0.311
Median intensity	2.264 [1.90;2.55]	3.467 [2.95;3.97]	**0.98**	**< 0.001**	+34.7% (1.2)	+33.7% (1.2)	2.336 [1.75;2.05]	2.255 [1.80;2.08]	0.579
Minimum intensity	1.877 [1.57;2.13]	2.868 [2.44;3.35]	**0.83**	**< 0.001**	+34.5% (1.0)	+33.4% (1.0)	1.955 [1.6;1.6002]	1.897 [1.6;1.601]	0.847
10th intensity percentile	1.936 [1.69;2.20]	2.955 [2.57;3.40]	**0.86**	**< 0.001**	+34.5% (1.0)	+34.5% (1.0)	2.015 [1.63;1.68]	1.958 [1.63;1.68]	0.452
90th intensity percentile	3.074 [2.15;3.63]	4.585 [6.39;5.44]	**0.94**	**< 0.001**	+33.0% (1.5)	+38.5% (1.7)	2.957 [1.97;3.09]	2.822 [2.19;2.94]	0.406
Maximum intensity	4.047 [2.55;5.11]	5.745 [4.07;7.13]	**0.84**	**0.032**	+29.6% (1.7)	+31.5% (0.4)	3.529 [2.14;4.26]	3.309 [2.42;4.06]	0.313
Intensity interquartile range	0.587 [0.22;0.79]	0.884 [0.45;1.20]	**0.67**	0.297	+33.6% (0.3)	+46.5% (0.4)	0.535 [0.19;0.70]	0.475 [0.33;0.62]	0.498
Intensity range	2.17 [0.68;3.40]	2.878 [1.36;4.24]	**0.60**	0.642	+24.6% (0.7)	+39.4% (1.0)	1.574 [0.54;2.65]	1.411 [0.82;2.46]	0.313
Intensity mean absolute deviation	0.364 [0.13;0.51]	0.517 [0.26;0.74]	**0.64**	0.446	+29.6% (0.2)	+46.1% (0.2)	0.301 [0.11;0.45]	0.27 [0.17;0.41]	0.394
Intensity-based robust mean absolute deviation	0.25 [0.09;0.34]	0.369 [0.19;0.50]	**0.65**	0.348	+32.3% (0.1)	+45.2% (0.1)	0.223 [0.08;0.30]	0.198 [0.12;0.27]	0.456

Intensity-based energy	8077.473 [731.2;11772.4]	16067.135 [2685.7;16389.0]	**0.68**	0.245	+49.7% (7989.7)	+63.9% (7785.5)	3987.985 [736.9;8000.3]	4001.366 [1320.6;4923.3]	0.602
Root mean square intensity	2.455 [1.93;2.88]	3.701 [3.15;4.12]	**0.99**	**< 0.001**	+33.7% (1.2)	+36.7% (1.4)	2.45 [1.78;2.29]	2.353 [1.87;2.21]	0.419
**Intensity histogram features (pyradiomics)**									
Discretized intensity entropy	3.35 [2.44;4.19]	4.027 [3.38;4.77]	**0.66**	0.293	+16.8% (0.7)	+20.7% (0.9)	3.201 [2.17;3.90]	3.229 [2.69;3.85]	0.589
Discretized intensity uniformity	0.157 [0.07;0.21]	0.093 [0.05;0.11]	**-0.69**	0.274	-68.3% (-0.06)	-82.5% (-0.05)	0.164 [0.09;0.25]	0.151 [0.09;0.19]	0.672
**Grey level co-occurrence based features (pyradiomics)**									
Joint maximum	0.071 [0.01;0.08]	0.033 [0.008;0.028]	**-0.71**	0.333	-113.0% (-0.04)	-193.7% (-0.02)	0.074 [0.02;0.10]	0.062 [0.02;0.07]	0.571
Joint average	25.289 [20.14;29.95]	38.029 [32.5;42.4]	**0.99**	**< 0.001**	+33.5 (12.7)	+31.6 (20.5)	25.55 [18.68;23.62]	24.524 [19.70;23.01]	0.448
Joint variance	38.384 [2.86;43.90]	59.455 [10.81;86.72]	**0.64**	0.729	+35.4% (21.1)	+64.6% (20.5)	22.296 [1.90;33.40]	15.298 [4.75;31.22]	0.293
Joint entropy	6.614 [5.12;8.04]	7.964 [6.81;9.36]	**0.69**	0.229	+17.0% (1.3)	+19.5% (1.6)	6.402 [4.63;7.85]	6.435 [5.65;7.64]	0.598
Difference average	2.749 [1.45;3.48]	4.098 [2.49;5.51]	**0.70**	0.092	+32.9% (1.3)	+52.6% (2.5)	2.777 [1.18;3.12]	2.511 [1.66;3.12]	0.513
Difference variance	8.138 [1.50;9.56]	14.893 [4.46;22.25]	**0.69**	0.207	45.4% (6.8)	+67.4% (8.8)	7.11 [0.99;8.11]	5.321 [2.06;7.53]	0.414
Difference entropy	2.773 [2.19;3.34]	3.364 [2.88;3.94]	**0.70**	0.122	+17.6% (0.6)	+25.0% (0.9)	2.751 [1.94;3.20]	2.695 [2.37;3.19]	0.563
Sum average	50.578 [40.28;59.9]	76.057 [65.06;84.77]	**0.99**	**< 0.001**	+33.5% (25.5)	+35.3% (27.2)	51.099 [37.37;47.25]	49.048 [39.40;46.02]	0.448
Sum entropy	4.297 [3.37;5.32]	5.042 [4.39;5.87]	**0.66**	0.300	+14.8% (0.7)	+17.7% (0.9)	4.14 [3.07;5.12]	4.16 [3.72;4.98]	0.603
Angular second moment	0.038 [0.006;0.34]	0.014 [0.002;0.012]	**-0.70**	0.298	-179.6% (-0.02)	-191.5% (-0.009)	0.039 [0.007;0.05]	0.032 [0.009;0.026]	0.570
Contrast	18.611 [3.59;22.89]	35.613 [10.7;52.9]	**0.69**	0.135	+47.7% (17.0)	+74.5% (26.5)	18.209 [2.38;17.66]	13.583 [4.83;17.29]	0.455
Dissimilarity	2.749 [1.45;3.48]	4.098 [2.49;5.5]	**0.70**	0.092	+32.9% (1.3)	+53.6% (2.5)	2.777 [1.18;3.12]	2.511 [0.38;0.51]	0.513
Inverse difference	0.447 [0.35;0.53]	0.354 [0.27;0.41]	**-0.70**	0.111	-26.1% (-0.1)	-45.6% (-0.1)	0.443 [0.38;0.57]	0.451 [0.38;0.51]	0.578

Normalized inverse difference	0.943 [0.93;0.96]	0.939 [0.93;0.94]	-0.32	0.860	-0.4% (-0.004)	-0.06% (-0.005)	0.936 [0.93;0.96]	0.938 [0.93;0.94]	0.958
Inverse difference moment	0.38 [0.26;0.47]	0.273 [0.18;0.33]	**-0.71**	0.120	-39.0% (-0.1)	-67.6% (-0.2)	0.374 [0.29;0.53]	0.382 [0.30;0.45]	0.612
Normalized inverse difference moment	0.993 [0.990;0.996]	0.992 [0.989;0.993]	-0.34	0.911	-0.1% (-0.001)	-0.1% (-0.001)	0.991 [0.991;0.996]	0.992 [0.989;0.993]	0.976
Inverse variance	0.347 [0.25;0.43]	0.265 [0.19;0.34]	**-0.70**	0.119	-30.9% (-0.1)	-63.2% (-0.1)	0.332 [0.28;0.45]	0.346 [0.29;0.36]	0.877
Correlation	0.504 [0.26;0.75]	0.564 [0.44;0.72]	0.40	0.875	+10.6% (0.1)	-4.9% (-0.03)	0.436 [0.25;0.75]	0.475 [0.44;0.72]	0.859
Autocorrelation	704.275 [414.9;909.0]	1546.75 [1060.3;1852.6]	**0.99**	**< 0.001**	+54.5% (842.5)	+59.8% (929.6)	742.051 [349.5;585.3]	651.082 [389.28;550.57]	0.400
Cluster tendency	134.925 [7.1;153.6]	202.207 [32.4;304.5]	**0.63**	0.832	+33.3% (67.3)	+68.3% (75.3)	70.975 [4.7;110.4]	47.609 [12.03;105.6]	0.280
Cluster shade	3521.348 [6.4;1980.4]	3618.007 [99.9;5143.1]	**0.59**	0.543	+2.7% (96.7)	+76.3% (696.5)	584.263 [6.04;1160.9]	269.613 [20.1;1097.3]	0.193
Cluster prominence	291266.819 [131.5;85341.4]	296546.876 [2738.9;288079.2]	**0.61**	0.497	+1.8% (5280.1)	+84.4% (31325.0)	44147.941 [61.51;42900.1]	12857.575 [385.7;40990.0]	0.199
Information correlation 1	-0.082 [-0.12;-0.03]	-0.082 [-0.11;0.05]	-0.34	0.552	-0.1% (0.0001)	-22.2% (0.02)	-0.063 [-0.12;-0.04]	-0.067 [-0.12;0.06]	0.809
Information correlation 2	0.603 [0.35;0.81]	0.672 [0.55;0.81]	**0.44**	0.876	+10.2% (0.07)	-3.1% (-0.02)	0.56 [0.34;0.81]	0.579 [0.52;0.79]	0.855
**Grey level run length based features (pyradiomics)**									
Short runs emphasis	0.913 [0.9;0.94]	0.94 [0.93;0.96]	**0.70**	0.074	+2.8% (0.03)	+3.4% (0.03)	0.912 [0.87;0.93]	0.902 [0.89;0.93]	0.295
Long runs emphasis	1.433 [1.25;1.54]	1.288 [1.17;1.31]	**-0.68**	0.079	-11.2% (-0.1)	-13.4% (-0.2)	1.455 [1.38;1.70]	1.559 [1.38;1.55]	0.143
Low grey level run emphasis	0.002 [0.001;0.003]	0.001 [0.0006;0.0011]	**-0.98**	**< 0.001**	-128.1% (-0.001)	-100.9% (-0.001)	0.002 [0.002;0.003]	0.0025 [0.0023;0.0028]	0.560
High grey level run emphasis	667.425 [399.4;852.9]	1466.873 [1019.9;1746.6]	**1.00**	**< 0.001**	+54.5% (799.4)	+59.2% (867.7)	695.531 [336.9;568.2]	615.382 [369.0;518.0]	0.398
Short run low grey level emphasis	0.002 [0.001;0.002]	0.001 [0.0006;0.001]	**-0.97**	**< 0.001**	-120.3% (-0.001)	-92.0% (-0.001)	0.002 [0.002;0.003]	0.0022 [0.0021;0.0025]	0.810
Short run high grey level emphasis	619.305 [364.1;801.7]	1388.529 [914.0;1674.4]	**0.99**	**< 0.001**	+55.4% (769.2)	+60.7% (845.6)	649.568 [290.0;517.9]	567.209 [330.2;484.1]	0.371

Long run low grey level emphasis	0.003 [0.002;0.004]	0.001 [0.0007;0.0014]	**-0.98**	**< 0.001**	-159.7% (-0.002)	-128.1% (-0.001)	0.003 [0.003;0.005]	0.004 [0.003;0.004]	0.212
Long run high grey level emphasis	901.439 [592.8;1073.9]	1836.455 [1372.4;2279.3]	**0.98**	**< 0.001**	+50.9% (935.0)	+51.6% (936.4)	919.992 [562.2;775.3]	870.743 [585.4;846.7]	0.930
Grey level non-uniformity	956.211 [336.2;1225.0]	704.603 [381.9;955.1]	0.15	0.280	-35.7% (-251.6)	+2.4% (16.2)	653.726 [361.3;1550.0]	809.671 [677.97;1493.35]	0.761
Normalized grey level non-uniformity	0.153 [0.07;0.21]	0.091 [0.05;0.11]	**-0.68**	0.275	-67.9% (-0.06)	-81.0% (-0.05)	0.159 [0.09;0.25]	0.148 [0.09;0.18]	0.709
Run length non-uniformity	9934.876 [1985.4;16236.5]	11323.826 [3832.5;13270.7]	**0.43**	0.768	+12.3% (1388.9)	+25.7% (2089.1)	4484.976 [1837.8;14936.2]	5145.396 [3103.6;11700.0]	0.627
Normalized run length non-uniformity	0.801 [0.77;0.86]	0.857 [0.83;0.91]	**0.71**	0.062	+6.5% (0.06)	+8.0% (0.06)	0.799 [0.71;0.82]	0.783 [0.75;0.83]	0.371
Run percentage	0.887 [0.86;0.93]	0.92 [0.91;0.95]	**0.70**	0.064	+3.6% (0.03)	+4.7% (0.04)	0.886 [0.82;0.90]	0.871 [0.86;0.90]	0.268
Grey level variance	37.036 [2.57;41.6]	57.474 [10.18;85.9]	**0.64**	0.739	+35.6% (20.4)	+68.1% (21.6)	21.545 [1.72;31.39]	14.805 [4.26;28.78]	0.297
Run length variance	0.152 [0.09;0.19]	0.101 [0.06;0.11]	**-0.65**	0.074	-50.3% (-0.1)	-43.4% (-0.04)	0.161 [0.14;0.24]	0.205 [0.13;0.19]	0.114
Run entropy	3.917 [3.0;4.77]	4.455 [3.85;5.08]	**0.62**	0.412	+12.1% (0.5)	+11.7% (0.5)	3.765 [2.88;4.56]	3.847 [3.31;4.39]	0.706
**Grey level size zone based features (pyradiomics)**									
Small zone emphasis	0.497 [0.43;0.59]	0.561 [0.49;0.66]	**0.57**	0.355	+11.5% (0.1)	+14.4% (0.1)	0.506 [0.39;0.55]	0.49 [0.36;0.50]	0.634
Large zone emphasis	434.516 [26.2;246.0]	65.513 [9.48;59.2]	**-0.59**	0.332	-563.2% (-369.0)	-264.8% (-55.5)	562.738 [62.62;820.51]	732.286 [88.41;541.77]	0.554
Low grey level emphasis	0.002 [0.001;0.003]	0.001 [0.0006;0.0011]	**-0.98**	**< 0.001**	-124.4% (-0.001)	-90.5% (-0.001)	0.002 [0.002;0.003]	0.002 [.002;0.003]	0.484
High grey level emphasis	723.418 [409.8;862.4]	1511.306 [997.0;1871.2]	**0.98**	**< 0.001**	+52.1% (787.9)	+59.5% (934.4)	695.486 [371.4;632.8]	611.782 [397.9;624.6]	0.336
Small zone low grey level emphasis	0.001 [0.0007;0.001]	0.005 [0.004;0.005]	**-0.83**	**< 0.001**	-97.1% (-0.0004)	-89.8% (-0.0004)	0.001 [0.0008;0.001]	0.001 [0.0009;0.001]	0.738

Small zone high grey level emphasis	393.079 [190.3;480.5]	898.586 [480.12;1262.8]	**0.92**	**< 0.001**	+56.3% (505.5)	+64.7% (571.0)	384.247 [146.5;349.6]	314.195 [127.2;295.8]	0.329
Large zone low grey level emphasis	1.427 [0.04;0.66]	0.068 [0.006;0.07]	**-0.74**	0.386	-2013.2% (-1.4)	-651.6% (-0.1)	1.782 [0.17;2.32]	2.368 [0.26;1.63]	0.534
Large zone high grey level emphasis	166847.021 [17237.6;108724.8]	75776.413 [16704.0;50415.7]	-0.30	0.565	-120.2% (-91070.6)	-89.0% (-18096.7)	188712.486 [32518.0;276018.1]	243079.958 [30860.9;251122.8]	0.570
Grey level non-uniformity	13.607 [6.62;23.5]	17.948 [9.99;23.96]	**0.50**	0.904	+24.2% (4.3)	+23.5% (3.1)	9.602 [3.57;16.77]	12.464 [4.55;14.70]	0.855
Grey level non-uniformity normalized	0.142 [0.05;0.20]	0.086 [0.04;0.11]	**-0.63**	0.320	-64.6% (-0.06)	-65.5% (-0.04)	0.159 [0.06;0.20]	0.178 [0.06;0.16]	0.418
Zone size non-uniformity	89.604 [4.23;142.2]	173.591 [25.1;165.4]	**0.62**	0.519	+48.4% (84.0)	+66.1% (59.8)	41.145 [2.24;64.34]	40.857 [4.03;47.4]	0.748
Zone size non-uniformity normalized	0.281 [0.21;0.34]	0.317 [0.24;0.41]	**0.47**	0.422	+11.4% (0.03)	+23.1% (0.1)	0.273 [0.19;0.32]	0.253 [0.18;0.27]	0.203
Zone percentage	0.238 [0.13;0.31]	0.355 [0.24;0.51]	**0.66**	**0.031**	+33.0% (0.1)	+4.5% (0.2)	0.251 [0.08;0.24]	0.215 [0.08;0.21]	0.413
Grey level variance	43.334 [3.93;52.85]	59.313 [10.82;86.9]	**0.60**	0.925	+26.9% (16.0)	+58.2% (21.6)	21.936 [2.82;34.05]	15.709 [5.40;32.15]	0.270
Zone size variance	373.77 [16.2;188.4]	46.277 [5.14;41.2]	**-0.54**	0.339	-707.7% (-327.5)	-241.8% (34.1)	470.34 [45.59;600.47]	626.375 [57.7;447.14]	0.639
Zone size entropy	5.082 [4.01;6.42]	5.774 [5.28;6.47]	**0.56**	0.546	+12.0% (0.7)	+10.5% (0.6)	4.742 [3.75;6.31]	4.784 [4.28;6.20]	0.630
**Neighborhood grey tone difference based features (pyradiomics)**									
Coarseness	0.045 [0.005;0.023]	0.014 [0.006;0.015]	-0.39	0.507	-233.0% (-0.03)	-41.1% (-0.004)	0.026 [0.005;0.02]	0.016 [0.006;0.014]	0.670
Contrast	0.155 [0.08;0.18]	0.234 [0.13;0.33]	**0.64**	**0.047**	+33.7% (0.08)	+46.3% (0.1)	0.211 [0.08;0.17]	0.165 [0.09;0.17]	0.537
Busyness	0.315 [0.17;0.35]	0.185 [0.10;0.28]	**-0.47**	0.057	-70.7% (-0.1)	-57.3% (-0.1)	0.351 [0.28;0.45]	0.448 [0.34;0.60]	0.608
Complexity	1125.952 [30.4;1092.6]	1890.278 [170.5;2466.2]	**0.65**	0.760	+40.4% (764.3)	+63.7% (383.0)	595.748 [15.3;668.5]	337.923 [48.5;617.9]	0.303
Strength	4.277 [0.76;4.81]	5.666 [1.40;9.81]	**0.53**	0.671	+24.5% (1.4)	+35.4% (1.3)	2.788 [0.58;3.03]	1.934 [0.67;3.14]	0.202

**Neighboring grey level dependence based on features (pyradiomics)**									
Low dependence emphasis	0.203 [0.12;0.27]	0.3 [0.21;0.43]	**0.69**	**0.030**	+32.6% (0.1)	+41.0% (0.1)	0.216 [0.08;0.21]	0.19 [0.09;0.20]	0.483
High dependence emphasis	20.758 [10.6;26.0]	13.169 [6.94;13.53]	**-0.65**	0.123	-57.6% (-7.6)	-89.5% (-8.7)	22.058 [17.1;33.0]	28.105 [16.51;26.27]	0.150
Low grey level count emphasis	0.002 [0.001;0.003]	0.001 [0.0006;0.001]	**-0.98**	**< 0.001**	-128.2% (-0.001)	-101.8% (-0.001)	0.002 [0.002;0.003]	0.0025 [0.0023;0.0028]	0.571
High grey level count emphasis	664.558 [392.8;856.2]	1464.499 [1022.6;1740.3]	**1.00**	**< 0.001**	+54.6% (799.9)	+59.3% (870.0)	696.485 [337.0;545.3]	616.463 [368.3;511.8]	0.408
Low dependence low grey level emphasis	0.0004 [0.0003;0.0005]	0.0002 [0.0001;0.0003]	-0.38	**0.002**	-46.7% (-0.001)	-42.0% (-0.0001)	0.0003 [0.002;0.0004]	0.0003 [0.0003;0.004]	0.613
Low dependence high grey level emphasis	171.444 [48.5;220.6]	504.486 [202.4;766.2]	**0.90**	**< 0.001**	+66.0% (333.0)	+72.6% (328.8)	201.906 [25.73;129.63]	148.213 [36.6;123.9]	0.378
High dependence low grey level emphasis	0.051 [0.02;0.06]	0.013 [0.004;0.15]	**-0.89**	**0.010**	-288.0% (-0.04)	-300.3% (-0.03)	0.061 [0.04;0.10]	0.079 [0.04;0.08]	0.170
High dependence high grey level emphasis	10909.861 [7907.2;12181.3]	16808.059 [10054.8;15042.0]	0.33	**0.008**	+35.1% (5898.2)	+1.2% (0.6)	10389.6 [7652.3;12187.6]	12358.47 [6639.9;11179.6]	0.131
Grey level non-uniformity	86.107 [29.5;106.3]	59.77 [33.9;80.6]	0.10	0.265	-44.1% (-26.3)	+1.2% (0.7)	60.982 [32.88;136.04]	77.792 [64.8;130.5]	0.828
Dependence count non-uniformity	178.905 [35.2;278.7]	245.986 [72.1;270.6]	**0.50**	0.893	+27.3% (67.1)	+35.2% (61.4)	89.502 [32.03;240.87]	97.189 [54.7;205.1]	0.670
Dependence count non-uniformity normalized	0.181 [0.13;0.21]	0.219 [0.19;0.26]	**0.59**	0.065	+17.5% (0.04)	+17.8% (0.03)	0.194 [0.13;0.20]	0.172 [0.14;0.18]	0.398
Grey level variance	36.56 [2.59;41.04]	57.37 [10.17;85.80]	**0.64**	0.721	+36.3% (20.8)	+68.7% (21.8)	21.602 [1.66;31.03]	14.827 [4.22;27.93]	0.304
Dependence count variance	4.004 [2.12;5.70]	2.78 [1.50;2.79]	**-0.50**	0.203	-44.0% (-1.2)	-45.6% (-1.0)	3.931 [2.58;6.36]	5.264 [3.13;4.95]	0.166
Dependence count entropy	5.668 [4.91;6.69]	6.191 [5.60;6.62]	**0.53**	0.642	+8.4% (0.5)	+7.3% (0.5)	5.484 [4.81;6.61]	5.695 [5.35;6.34]	0.911

p-value for the predictive value of carbidopa premedication in a linear regression model also including the histo-molecular diagnosis without and with Tumor to normal Brain ratios (TBR) (in bold. significant p-values). Absolute correlation coefficients in bold are >=0.4. TTP: Time to Peak; SUV: Standardized Uptake Value

Table S2−S4: please see the excel files

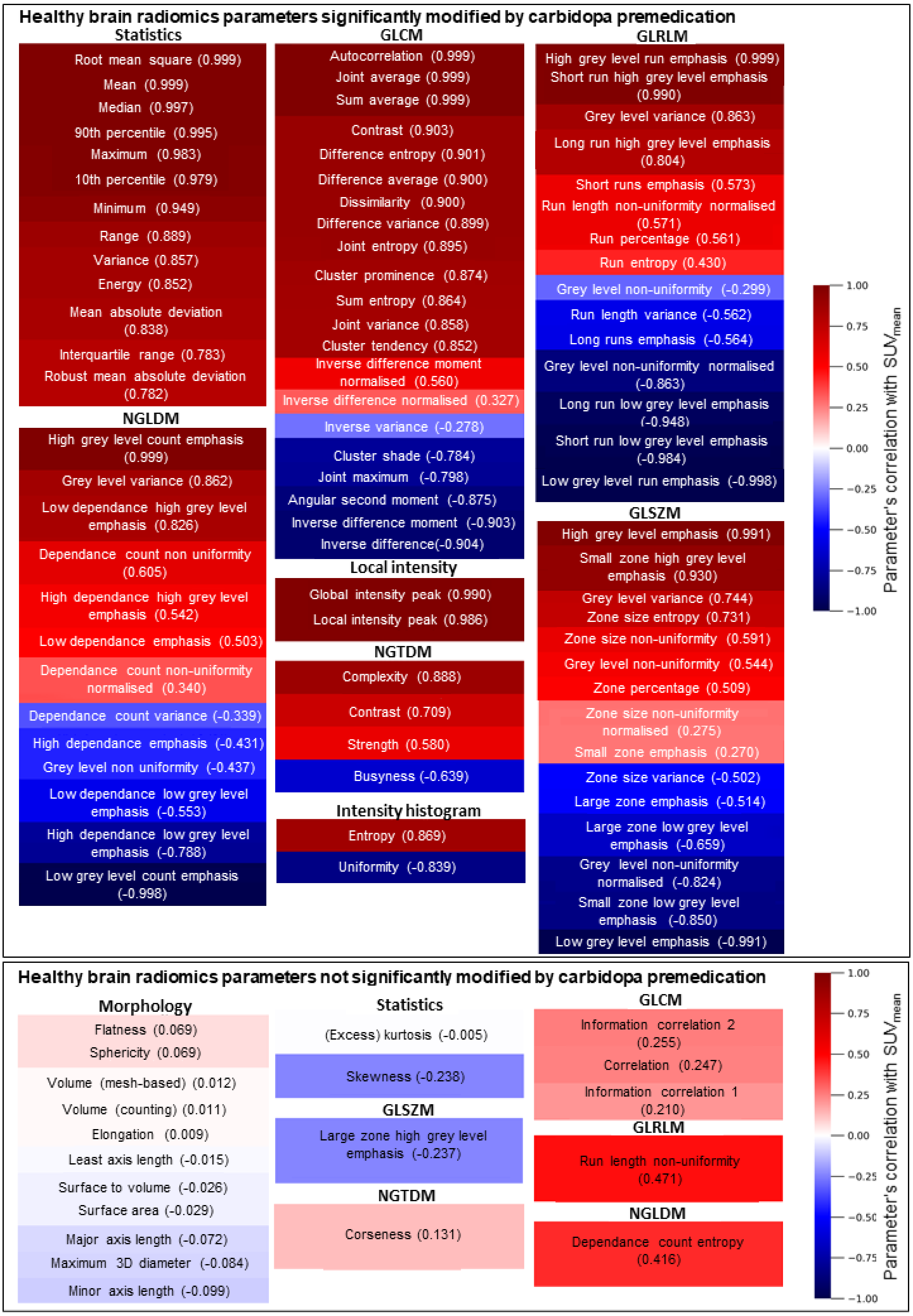

Figure S1. Effect of carbidopa premedication on healthy brain radiomics parameters.

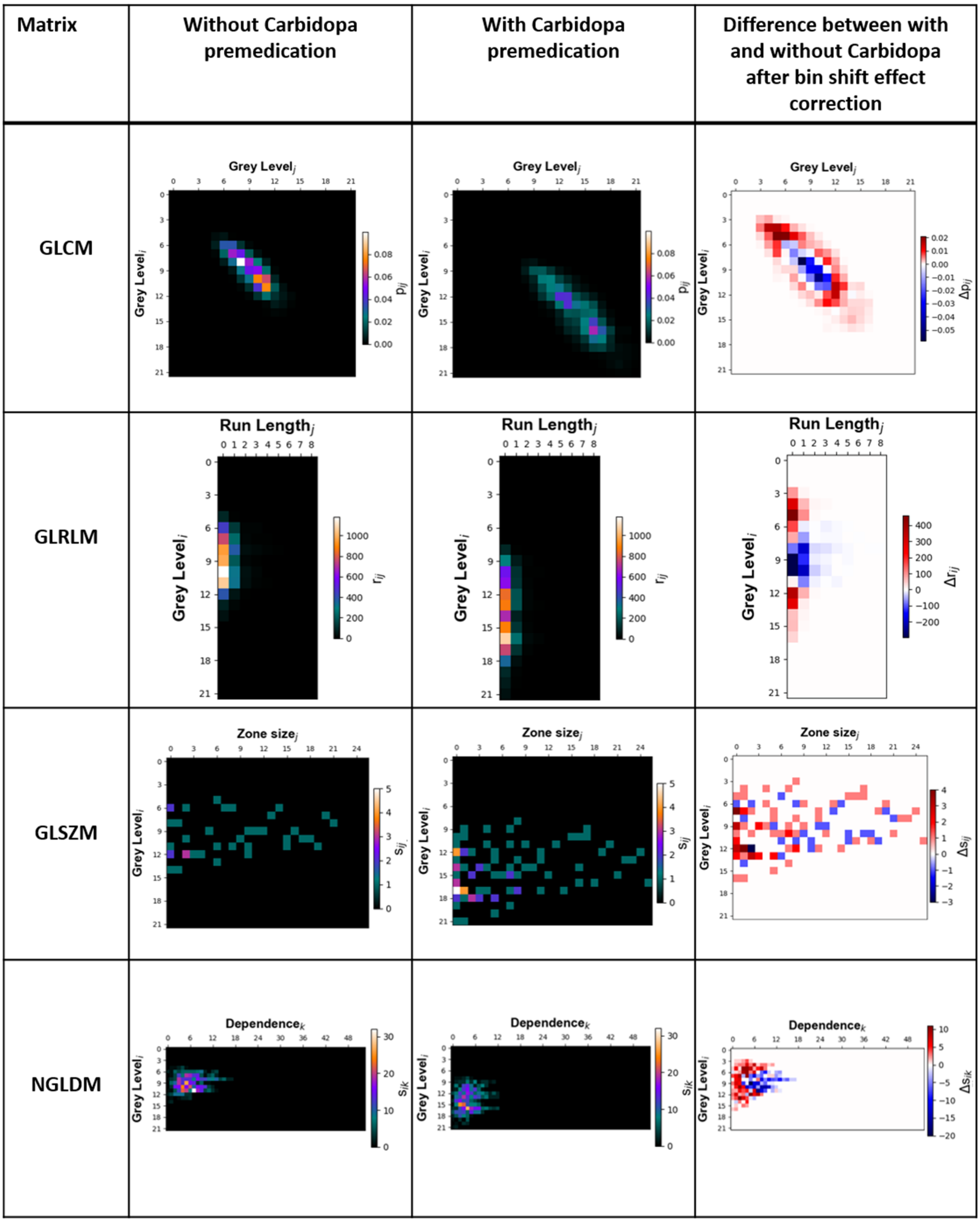

Figure S2. Illustration of bin spread effect on matrices in which some textural features were significantly modified with carbidopa, but poorly correlated to SUVmean. These four textural matrices are: 1. Grey level co-occurrence matrix (GLCM) with modified parameters: Inverse difference normalized, Inverse variance; 2. Grey level run length matrix (GLRLM) with modified parameter: Grey level non-uniformity; 3. Grey level size zone matrix (GLSZM) with modified parameters: Small zone emphasis, Zone size non-uniformity normalized; 4. Neighboring grey level dependence based matrix (NGLDM) with modified parameters: Dependence count non-uniformity normalized, and Dependence count variance. These textural matrices were extracted from a static healthy brain image of a patient without carbidopa

premedication and a similar static image was then generated to simulate the effect of carbidopa premedication. This simulation was performed by multiplying by 1.5 the voxel-values in the original static image (to reproduce the 50% increase in patients, reported in our current study). To emphasize the bin spread effect of carbidopa premedication on these matrices, the differences between carbidopa premedication and no premedication were computed after correcting matrices for the bin shift (linked to the increase of SUV values). pij represent the joint probability for the combination of grey levels i and j in neighboring voxels of Cancers 2021, 13, x FOR PEER REVIEW 8 of 8 the image. rij correspond to the joint probability of the number of runs with grey level i and length j in the image. sij represent the number of zones with gray level i and size j appearing in the image. sik is the joint probability for a voxel with grey level i and with k dependent on voxels appearing in the image.
